# Novel Applications for Oxalate-Phosphate-Amine Metal-Organic-Frameworks (OPA-MOFs): Can an Iron-Based OPA-MOF Be Used as Slow-Release Fertilizer?

**DOI:** 10.1371/journal.pone.0144169

**Published:** 2015-12-03

**Authors:** Manuela Anstoetz, Terry J. Rose, Malcolm W. Clark, Lachlan H. Yee, Carolyn A. Raymond, Tony Vancov

**Affiliations:** 1 School of Environment, Science and Engineering, Southern Cross University, Lismore, NSW, 2480, Australia; 2 Marine Ecology Research Centre, School of Environment, Science and Engineering, Southern Cross University, Lismore, NSW, 2480, Australia; 3 Southern Cross Plant Science, Southern Cross University, Lismore, NSW, 2480, Australia; 4 NSW Department of Primary Industries, Wollongbar Primary Industries Institute, Wollongbar, NSW, 2477, Australia; North Carolina State University, UNITED STATES

## Abstract

A porous iron-based oxalate-phosphate-amine metal-organic framework material (OPA-MOF) was investigated as a microbially-induced slow-release nitrogen (N) and phosphorus (P) fertilizer. Seedling growth, grain yields, nutrient uptake of wheat plants, and soil dynamics in incubated soil, were investigated using OPA-MOF vs standard P (triple-superphosphate) and N (urea) fertilizers in an acidic Ferralsol at two application rates (equivalent 120 and 40 kg N ha^-1^). While urea hydrolysis in the OPA-MOF treatment was rapid, conversion of ammonium to nitrate was significantly inhibited compared to urea treatment. Reduced wheat growth in OPA-MOF treatments was not caused by N-deficiency, but by limited P-bioavailability. Two likely reasons were slow P-mobilisation from the OPA-MOF or rapid P-binding in the acid soil. P-uptake and yield in OPA-MOF treatments were significantly higher than in nil-P controls, but significantly lower than in conventionally-fertilised plants. OPA-MOF showed potential as enhanced efficiency N fertilizer. However, as P-bioavailability was insufficient to meet plant demands, further work should determine if P-availability may be enhanced in alkaline soils, or whether central ions other than Fe, forming the inorganic metal-P framework in the MOF, may act as a more effective P-source in acid soils.

## Introduction

Crop yields increased significantly after the green revolution due to the application of fertilizers, and breeding of fertilizer-responsive crop varieties. The two nutrients that tend to be most limiting for crops are nitrogen (N) and phosphorus (P). N- and P-fertilizer applications are frequently required to sustain high crop yields[1]. As such, the demand for N- and P- fertilizers has increased dramatically over recent decades and is projected to increase further over the coming decade [2]. Exacerbating the strong N-fertilizer demand is the inefficient uptake of fertilizer-N by most crops; major grain crops only recover around a third of the fertilizer-N applied in the year of application [3]. Although some N remains in the soil and in crop residues that is available to subsequent crops, N losses from the system can be environmentally problematic, contributing to agricultural greenhouse gas emissions (N_2_O) and eutrophication of water bodies [4, 5]. Similarly, efficient P-fertilizer use is also low, predominantly due to soil P fixation, such that only a proportion of the P added as fertilizer is ever taken up by crops [6].

Nitrogen and P nutrient losses occur mostly because of incongruence between N and/or P application timing and plant demand [1]. Fertilizer-P is typically applied to wheat crops at sowing, but the strongest P-demand tends to occur from tillering to anthesis [7, 8]. Thus, much of the water-soluble P-fertilizer applied at sowing is not taken up during the first month after sowing by wheat crops, and reacts with soil minerals to become ‘fixed’. In addition, N uptake by wheat is somewhat driven by the soil supply, but the pattern of N accumulation by wheat crops is not dissimilar from dry matter accumulation, with large N demands from tillering to mid grain filling [1]. Hence, the application of rapid-release N-fertilizer (e.g. urea) pre-sowing or at sowing provides significant opportunity for gaseous N losses (N_2_O, NH_3_) and N-leaching [9].

An agronomic option that can better match nutrient supply with crop demand is to use slow release fertilizers or amendments. For example, nitrification inhibitors which slow the rate of NH_4_^+^ conversion to NO_3_^-^ have been shown to reduce N losses as N_2_O [10]. Another novel option to slow plant nutrient release is to use bacterial processing (assisted mineralisation) to accomplish fertilizer compound breakdown. Bacterial processing delays nutritional element release, as bacterial populations require time to accrue and adjust. A prerequisite for bacterial-assisted mineralisation is sufficient solubility of an activating carbon source to stimulate the soil microorganisms. Oxalate is such a carbon source because soils generally harbour oxalotrophic bacteria that may derive all their energy needs from oxalates via the oxalate-carbonate pathway. However, to stimulate oxalotrophic bacteria, soluble oxalate concentrations in soil need to be at least 1 mgL^-1^ [11]. Moreover, microbial oxalate consumption via the oxalate-carbonate pathway mineralises and releases carbonates into the soil environment, resulting in increased soil-pH [12].

Oxalate is a low molecular weight organic acid (LMW-OA) and plays central roles in the interactions between soil microorganisms, plants and soil including provision of protection against grazing when incorporated into plants, or enabling chelation of soil minerals and exchange processes thereof [13]. Plants and other organisms actively release LMW-OAs into the rhizosphere to increase P-bioavailability [14]; and subject to soil type, oxalate has been found to be one of the more effective LMW-OAs [15, 16]. Despite the quantities of oxalate produced by plants, few oxalate minerals persist in soils because of the bioprocessing, and they tend to build up in isolated niches [17, 18].

Oxalate also plays an important role in the material sciences as an organic ligand for the synthesis of metal-organic framework materials (herein referred to as MOFs). MOFs are a group of minerals characterised by porous physical frameworks (as a result of their chemical structures) which have been continuously developed over the last few decades to satisfy the strong demand for new types of catalysts, molecular sieves, gas storage materials, medical drug carriers, and environmental remediation ameliorants [19, 20]. Their frameworks consist of layers of inorganic polyhedra, such as ferric/ferrous oxides and phosphoric oxides interconnected by organic ligands, such as simple carboxylates, or complex ring-structures. One commonly used carboxylate is oxalate, which readily substitutes for phosphate while showing strong coordination tendencies particularly with transition metals [21]. Oxalate as an organic ligand is particularly favoured because of its ability to connect not only in-plane, but also out-of-plane, substantially increasing the variability in pore sizes in the resulting two-or three dimensional networks [22]. Overall, oxalate-based MOFs predominantly have an anionic framework that is neutralised by cationic guest molecules residing inside the pores, which are typically the structure-directing agents (SDA, or “template”) used during synthesis, such as di-amines. However, neutral frameworks have been found where the pore-residing guest molecule does not have to fulfil a charge-neutralising role [23]. A MOF with the organic ligand oxalate (“O”), containing iron-phosphates (phosphate “P”) combined with SDA, urea (di-amine “A”, hence the combination known as OPA), contains the plant nutritional elements N and P, plus the additional micronutrient element iron (Fe), and thus P as an integral part of the framework structure, and N as pore-residing urea.

**Fig 1:**
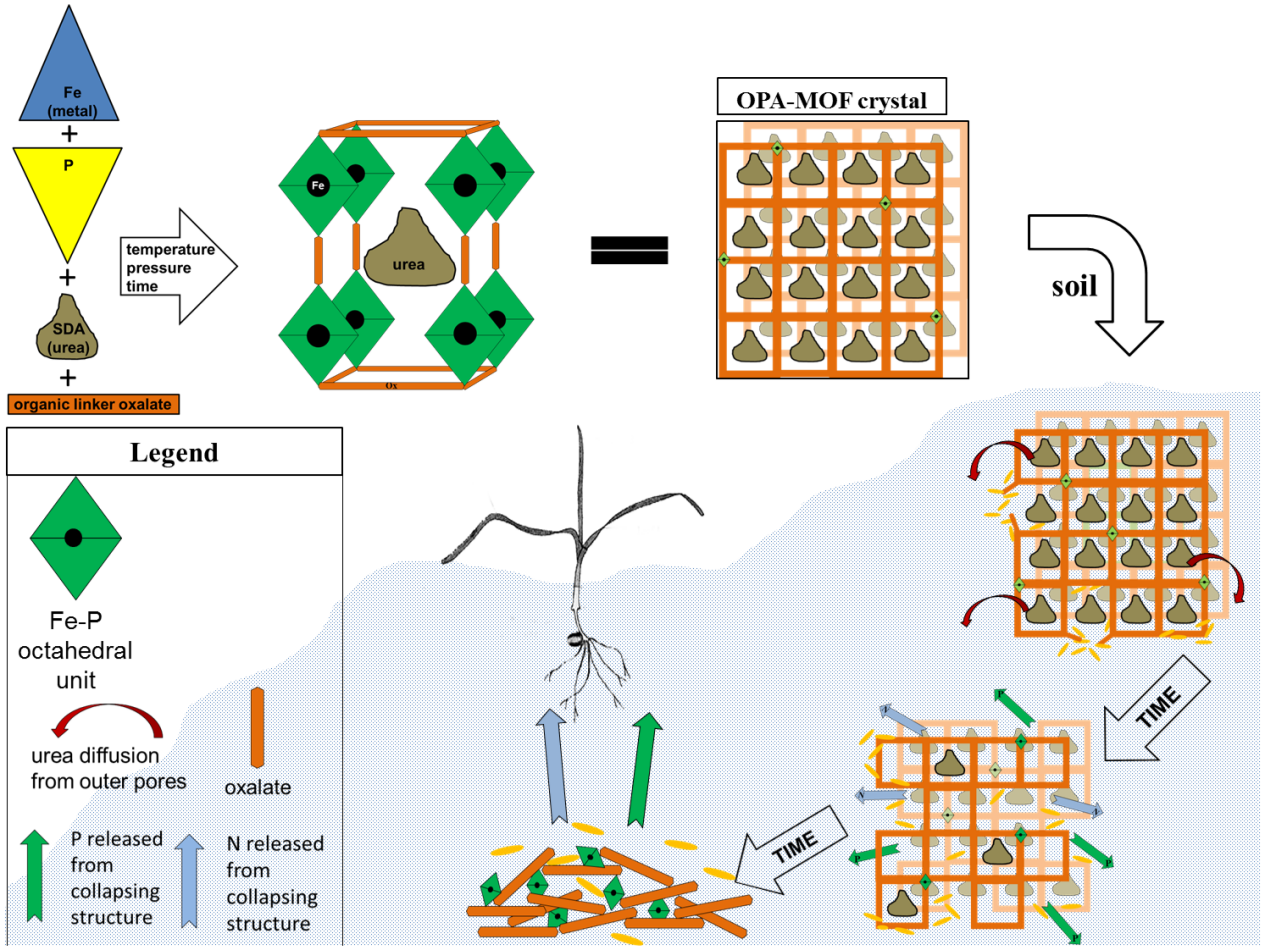
Conceptual Diagram. The conceptual diagram describes OPA-MOF synthesis, and bacterial processing and mineralisation of the structurally incorporated oxalate when applied to soil; plant nutrient release is proposed as a result of the microbially induced structural collapse of the mineral, where N is delivered from pore-residing guest molecule urea and P from the Fe-P octahedral units that form the framework.

In this study, we investigate the potential of the urea-templated oxalate-phosphate-amine MOF (OPA-MOF) as a novel slow-release fertilizer. We hypothesise that the OPA-MOF has a slow-release fertilizing potential for crops grown on acidic soils, where microbial consumption of the oxalate structural organic linker drives the collapse of the framework structure, thereby releasing the Fe-phosphate from within. Moreover, the microbially-mediated oxalate breakdown will increase pH in the soil from carbonate mineralisation via the oxalate-carbonate pathway, aiding the pH-dependent mobilisation of the P bound to Fe-oxides and hydroxides. The synthesis and structure, as well as the hypothesised bacterial breakdown of OPA-MOF are schematically represented in Fig 1.

## Materials and Methods

### Soil material

Soil from the top 150-mm horizon of a P-deficient rhodic Ferralsol [24] was collected at New South Wales Department of Primary Industries Wollongbar Agricultural Institute (28°50′S, 153°25′E) in north-eastern NSW, Australia. Soil was air-dried to ~ 25% moisture and sieved to 4 mm. A subsample was dried at 65°C for 3 days and analysed for physico-chemical properties and soil nutrient status (Table 1). Analyses for Bray I and II, nitrate-N, ammonium-N, K, Mg and Ca, DTPA-extractable micronutrients, ECEC, electrical conductivity and soil pH were undertaken using standard methods as described by Forster [25] and Rayment and Lyons [26] at the NATA accredited Environmental Analysis Laboratory (EAL) at Southern Cross University (SCU), Lismore, NSW, Australia. Briefly, soil pH was measured in water (1:5), and total N and C were measured by combustion using a LECO TruMAC CNS analyzer. Extractable cations were quantified using inductively coupled plasma optical emission spectroscopy (ICP-OES 4300D, Perkin Elmer, USA). Concentrations of extracted nitrate- and ammonium-N were quantified using a flow injection analyser (FIA) after KCl extraction.

**Table 1:** Experimental soil parameters.

**Parameters and Macronutrients**	**pH**_**water**_	**Conductivity**	**ECEC**	**Nitrate-N**	**NH**_**4**_ **-N**	**Bray I-P**	**Bray II-P**
		(dS m ^-1^)	(cmol+kg^-1^)	(mg kg^-1^)	(mg kg^-1^)	(mg kg^-1^)	(mg kg^-1^)
	5.88	0.068	7.74	13.6	6.0	1.4	10
**Macro- and Micronutrients**	**Ca**_**available**_	**Mg**_**available**_	**K**_**available**_	**Zn**	**Mn**	**Fe**	**S**
	(mg kg^-1^)	(mg kg^-1^)	(mg kg^-1^)	(mg kg^-1^)	(mg kg^-1^)	(mg kg^-1^)	(mg kg^-1^)
	854	94	59	1.7	20	63	17.8

Soil physico-chemical parameters and nutrient concentrations of experimental soils (Ferralsol) used for all trials.

### Fertilizer material

Fertilizer combination treatments were made from commercially-available agricultural fertilizers with urea as the N source and triple-superphosphate as the P source in a pelletised form. Commercially-available calcium-oxalate was used as an oxalate source (Ox). The oxalate-phosphate-amine metal-organic-framework mineral (OPA-MOF) was synthesised from commercially available chemicals. Briefly, a homogenized starter solution made from ferric chloride, orthophosphoric acid, oxalic acid, urea, and water, mixed to a molar ratio of 1:6:1:3:100, respectively, was filled into Teflon^®^ coated polypropylene (PP) flasks and locked into steel pressure-digestion vessels. The hydrothermal reaction took place at 100°C over 24 hours, and precipitated the crystalline OPA-MOF mineral. After filtering and drying to constant weight the mineral was ground to a powder using an agate mortar and pestle, and passed through a 0.5 mm sieve before application. In all there were eight fertilizer treatments and a nil fertilizer control (Table 2). The eight fertilizer treatments were applied at two rates, high and low (Table 2), where the dose rates were 120 kg Nha^-1^ and 40 kg Nha^-1^, respectively, on the basis of N concentration of the synthesised OPA-MOF (3.1%). The required amounts for the two rates of N application were calculated for OPA-MOF, and then the required amounts of urea, superphosphate and oxalate back-calculated on the basis of the respective mineral concentrations in OPA-MOF (12.5% P; 14.5% Ox).

**Table 2:** Experimental nutrient applications.

Treatment	N	P	Ca	Oxalate
**Control**	nil	nil	nil	nil
***High rate***				
**N**	120	-	-	-
**P**	-	350	225	-
**Ca-Ox**	-	-	269	589
**OPA-MOF**	120	350	-	589
**N+Ox**	120	-	269	589
**P+Ox**	-	350	449	589
**N+P**	120	350	225	-
**N+P+Ox**	120	350	449	589
***Low rate***				
**N**	40	-	-	-
**P**		120	78	-
**Ca-Ox**	-	-	93	205
**OPA-MOF**	40	120	-	205
**N+Ox**	40	-	93	205
**P+Ox**	-	120	171	205
**N+P**	40	120	78	-
**N+P+Ox**	40	120	171	205

Treatments and elemental nutrient amounts applied in the pot trial, given as equivalents of kgha^-1^ at two rates; treatments were made from individual nutrient sources (urea as N-source, superphosphate as P-source, calcium oxalate as oxalate source), or of combinations thereof; OPA-MOF was only used individually.

### Experiment 1—Crop growth using OPA-MOF as a nitrogen and phosphorus fertilizer

A pot study was conducted to investigate the impact of the synthesised OPA-MOF compared with traditional N- and P-fertilizers on the growth, nutrient uptake and grain yield of wheat (*Triticum aestivum* L.). The conventional N and P fertilisers were urea (46% N) and triple superphosphate (20.6% P, 17% Ca), respectively. The experiment comprised 17 fertilizer treatments of varied combinations (8 fertilizer treatments x 2 fertilizer rates plus a nil fertilizer control) with four replicates. To enable measurement of biomass and nutrient uptake during early growth as well as at maturity, a duplicate set of plants were grown, so in total there were 136 pots (17 fertilizer treatments x 2 harvest dates x 4 replicates). A fully randomised block design was employed, with pots laid out randomly in each block and re-randomised weekly. The experiment was conducted in a naturally lit, temperature-controlled glasshouse at Southern Cross University, Lismore, NSW, Australia; temperature was maintained to a daytime maximum of 30°C.

Soil was added at a moisture content of 27.5% (w/w) into black, free-draining pots of two different sizes: 15-cm-diameter pots (500 g dry soil equivalent) were used for the harvest of wheat seedlings at 6 weeks after sowing (WAS); 30-cm-diameter pots (7500 g dry soil equivalent) were used to grow the wheat plants to maturity. All fertilizers were applied to the soil surface and immediately watered in prior to sowing. The day after fertilizer applications, pots were sown with five seeds of wheat cv. Dollarbird at 15 mm depth. At 10 days after sowing (DAS) plants were thinned to two evenly-sized seedlings per pot. All watering was done manually with tap water (pH 6.8) in small doses every alternate day to avoid leaching and nutrient losses. Once per week soil moisture was restored to ~ 75% (w/w) by weighing pots and adding the appropriate amount of water.

Plants in the small pots were grown until the most advanced plants reached Zadoks growth stage GS24 [27], approximately 6 WAS. Plants were harvested by severing the shoots at the soil surface. Shoots were then oven-dried to the point of stable mass and finely ground. Plants in the large pots were cultivated to maturity (17 WAS) and harvested as above. Mature shoots were separated into grain and straw tissue, and dried, weighed and ground as above. A subsample of the ground tissues was digested with nitric acid in a MARS microwave oven (CEM Corp., Matthews, NC, USA). The concentrations of P, S, Ca, Mg, K, Cu, Mn and Zn in the digest were determined by inductively coupled plasma mass spectroscopy (ICP-MS) (Perkin Elmer Optima 4300). Tissue nutrient contents were determined by multiplying the tissue nutrient concentrations by the respective tissue dry weights.

### Experiment 2—Soil incubation study assessing nutrient release dynamics from fertilizers

A soil incubation experiment was conducted simultaneously with the pot trial to assess nutrient release over time from OPA-MOF and control fertilizers. The experimental design was a randomised block consisting of eight fertilizer treatments at high/low rates (as experiment 1) x five sampling time-points (t_4 weeks_, t_float1_, t_float2_, t_harvest_ and t_post-harvest_) with three replicate pots for sampling time and combination (120 pots for high).

Soil (as above) was added at 54 g dry soil equivalent per pot to 5-cm-diameter pots and the moisture adjusted to 38% (w/w). Soils were incubated in a shade-house at the School of Environment, Science and Engineering at SCU, Australia. Pots were loosely covered with a clear polyvinyl chloride sheet to minimise evaporation of soil moisture whilst providing some protection against the elements. Sampling times after the initial sampling at 4 WAS were based on the development of wheat in the parallel wheat growth study (Experiment 1). The 2^nd^ sampling t_float1_ was undertaken in week 7, close to harvesting of the 6 WAS-seedlings in experiment 1, t_float2_ occurred at week 13, T_harvest_ occurred at 17 weeks and t_post-harvest_ occurred at 22 weeks. Immediately after each sampling, the soil was thoroughly homogenised and sent to EAL for analysis of available and extractable macronutrients (Bray I and II, nitrate-N, ammonium-N, K, Mg and Ca), DTPA-extractable micronutrients, ECEC, conductivity, and pH using methods as described by Forster [25] and Rayment and Lyons [26]. Ambient temperatures over the duration of the incubation study ranged from a minimum of 4.8°C at night to a maximum of 38.7°C during the day.

### Statistical analyses

All statistical analyses were performed using GENSTAT 16.1 software [28]. ANOVAs were performed on each data set to test for significant differences between the applied treatments. A 2-way-ANOVA was used to test for differences between the applied treatments and the application level. Where appropriate, data were transformed to satisfy normality assumptions. Duncan’s Test was performed to compare the treatments with each other (including controls) for each level of treatment (high/low) separately. For analysis of soil samples, 2-way-ANOVA and Duncan’s Tests were performed for individual sampling time points to allow comparison between treatments, and for individual treatments to allow comparison between the different sampling time points.

## Results

The high and low fertilizer application rates (Table 2) showed similar responses, hence, for simplicity only the high-rate fertilizer application results are presented in detail. Low fertilizer application rate data are provided in S1 and S2 Tables.

### Experiment 1: Wheat growth study

#### Biomass production at 6 weeks after sowing

Plants grown in the absence of N- or P-fertilizer did not tiller (data not presented) and produced only 175 g of biomass pot^-1^ at 6 WAS (Table 3). Phosphorus was the most limiting nutrient in the highly P-fixing soil, since biomass increased significantly (~ 3-fold) with P-fertilizer addition in the absence of N, but no growth increase was observed in plants that received N in the absence of P (Table 3). In treatments where both N and P were applied, plants produced 6–7 tillers and a 4-fold biomass increase compared to nil-fertilizer pots. Applications of Ca-Ox either alone or in combination with N or P treatments did not yield significant biomass improvements above the relative controls (Table 3). Treatments receiving OPA-MOF resulted in significantly (p ≤ 0.05) higher biomass production than the control and N treatments in the absence of P (274 g pot^-1^ vs 174 g pot^-1^ and 194g pot^-1^, respectively). However, OPA-MOF produced significantly (p ≤ 0.05) less biomass than P-only treatments (500 g pot^-1^), and only around 25–30% of the biomass for combined (N+P) treatments (Table 3).

**Table 3:** Nutrients in shoot tissues from high treatment rate.

**Treatment**	**Biomass**	**Macronutrient concentration**					**Micronutrient concentration**		
**a)**	(mg pot^-1^)	(%)					(mg kg^-1^)		
		**N**	**P**	**K**	**Ca**	**Mg**	**Cu**	**Mn**	**Zn**
*Control*	*174*.*5 A*	*3*.*66 c*	*0*.*15 ab*	*5*.*07 e*	*0*.*71 bc*	*0*.*27 b*	*12*.*0 c*	*88 c*	*38 c*
N	194.3 A	4.20 d	0.13 a	4.33 d	0.86 d	0.26 ab	9.9 b	268 g	45 d
Ca-Ox	202.8 A	3.39 b	0.16 b	4.93 e	0.65 ab	0.24 ab	8.5 ab	74 b	31 b
N+Ca-Ox	206.5 A	4.42 e	0.17 b	4.86 e	0.94 e	0.27 ab	9.9 b	208 f	49 e
**OPA-MOF**	**274.2 B**	**5.30 f**	**0.25 c**	**4.43.e**	**0.63 a**	**0.26 ab**	**9.7 b**	**184 e**	**53 f**
P	503.2 C	2.34 a	0.59 d	3.63 c	0.72 c	0.26 ab	7.9 b	84 bc	23 a
P+Ca-Ox	507.2 C	2.25 a	0.55 d	3.58 c	0.68 abc	0.23 a	8.2 a	61 a	21 a
N+P	805.5 D	6.18 h	0.79 e	2.53 b	1.29 g	0.61 c	9.5 ab	138 d	39 c
N+P+Ca-Ox	1016.5 D	5.81 g	0.75 e	2.08 a	1.21 f	0.57 c	9.9 b	74 b	38 c
	**Biomass**	**Macronutrient content**					**Micronutrient content**		
**b)**	(mg pot^-1^)	(mg pot^-1^)					(μg pot^-1^)		
		**N**	**P**	**K**	**Ca**	**Mg**	**Cu**	**Mn**	**Zn**
*Control*	*174*.*5 A*	*6*.*39 A*	*0*.*26 AB*	*8*.*85 A*	*1*.*24 A*	*0*.*47 A*	*2*.*09 AB*	*15*.*36 A*	*6*.*63 AB*
N	194.3 A	8.16 AB	0.21 A	8.41 A	1.67 BC	0.51 A	1.94 A	52.06 C	8.74 BC
Ca-Ox	202.8 A	6.87 A	0.32 BC	10.00 AB	1.32 AB	0.49 A	1.83 A	15.00 A	6.29 A
N+Ca-Ox	206.5 A	9.13 BC	0.35 C	10.04 AB	1.94 C	0.56 AB	2.07 AB	42.95 C	10.12 C
**OPA-MOF**	**274.2 B**	**14.55 D**	**0.69 D**	**12.12 B**	**1.71 C**	**0.71 B**	**2.74 B**	**50.32 C**	**14.54 D**
P	503.2 C	11.78 CD	2.95 E	18.27 C	3.62 D	1.31 C	5.29 C	42.53 C	11.33 CD
P+Ca-Ox	507.2 C	11.45 CD	2.82 E	18.20 C	3.45 D	1.17 C	4.07 C	30.90 B	10.67 C
N+P	805.5 D	49.63 E	6.36 F	19.95 C	10.45 E	4.95 D	7.71 D	113.37 E	30.76 E
N+P+Ca-Ox	1016.5 D	58.75 E	7.56 F	20.34 C	12.24 E	5.87 D	10.07 D	73.29 D	38.67 E

Macro- and micronutrient concentrations (part a) and -contents (part b) in shoot tissue of six-week-old wheat plants from high application rate of various fertilizers. Mean values followed by the same letter are not significantly different at the 5% confidence level. Upper case letters in part b (contents) and for biomass are from analysis of ln-transformed data; ANOVA with n = 4 replicates.

#### Shoot nutrient concentrations and content at 6 weeks after sowing

Applications of N in the absence of P significantly (p ≤ 0.05) increased shoot N concentrations, with a further small but significant (p ≤ 0.05) increase in N concentration when Ca-Ox was applied in combination with N (Table 3). Further, all N treatments resulted in significantly increased (p ≤ 0.05) shoot Mn concentrations compared to all other similar treatments without N (Table 3) suggesting there may be some redox response resulting in Mn reduction through urea oxidation.

Application of P without N more than doubled the shoot content of all other nutrients except N (also a limiting nutrient in this soil) and Zn (Table 3). However, most increases in nutrient content were concomitant with increased biomass production rather than some specific effect, since shoot concentrations of nutrients remained stable (Ca, Mg) or even declined slightly (N, K, Cu, Zn) in all P treatments (Table 3). The additional supply of Ca-Ox with P had little effect on nutrient or trace element concentrations and contents except of Cu (increase) and Mn (decrease). In contrast to P-alone, N+P additions to the soil led to an increase in shoot N, Mg, Ca and Mn concentrations compared to the controls, while shoot Zn and Cu did not differ significantly (Table 3). Notably, shoot K concentrations significantly (p ≤ 0.05) declined for N+P treatments compared to the nil control. Interestingly, Ca-Oxalate additions to the N+P treatment caused a significant (p ≤ 0.05) reduction in shoot N, K, Ca and Mn concentrations compared to N+P treatment alone (Table 3), but these changes did not influence micronutrient contents, except Mn, which declined significantly. Ca-Ox inclusion with any P treatment failed to improve shoot Ca concentrations compared to P treatments only, suggesting that soil Ca supply was not limiting.

Treatments with OPA-MOF significantly (p ≤ 0.05) increased the shoot contents of all nutrients except Cu compared to control treatments, but shoot contents for most nutrients in the OPA-MOF treatment were significantly lower than those where any P was added. Shoot N-contents from OPA-MOF treatment, however, were not significantly different to those where P was provided. Similarly, Zn and Mn micronutrient contents from OPA-MOF soils increased significantly compared to controls and were not significantly different to treatments receiving P alone (Table 3). Increased shoot nutrient contents of K, Mg, Ca and Cu from MOF application were concomitant with increased biomass production, since the concentrations of nutrients remained unchanged (K, Mg) or decreased slightly (Ca, Cu) compared to control. However, shoot N, P, Mn and Zn contents and concentrations were significantly higher than in control plants.

#### Grain yields at maturity

Like the seedling biomass yields, the highest grain yields (~ 19 g pot^-1^) were obtained when both N- and P-fertilizers were added, compared to 3 g pot^-1^ for controls (Table 4). N-only additions led to a small but significant increase in grain yield compared to controls while P addition increased grain yields 3-fold (Table 4). Ca-Oxalate additions to N, P, or N+P treatments failed to demonstrate significant yield increases above their counterpart treatments without Ca-Oxalate (Table 4), and caused a small but significant (p ≤ 0.05) decline in grain yields in the N+Ca-Ox treatment compared to the N-only treatment (Table 4). OPA-MOF treatment resulted in a significant (doubling) of grain yields compared to the nil control and all other treatments which did not receive P (Table 4). Any changes in grain yields among treatments were a result of grain number, as the “1000-grain” weight did not significantly differ among treatments (data not shown).

**Table 4:** Nutrients in grains from high treatment rate.

**Treatment**	**Yield**	**Macronutrient concentration**					**Micronutrient concentration**		
**a)**	(g pot^-1^)	(%)					(mg kg^-1^)		
		**N**	**P**	**K**	**Ca**	**Mg**	**Cu**	**Mn**	**Zn**
Control	3.00 ab	3.43 a	0.50 ab	0.70 a	ns	0.18 a	7.7 ab	29 b	60 a
N	4.40 c	3.22 a	0.40 def	0.62 b	ns	0.15 b	7.0 ab	39 a	55 ab
Ca-Ox	3.84 bc	3.43 a	0.47 abc	0.62 ab	ns	0.17 ab	7.3 ab	29 b	55 ab
N+Ca-Ox	2.85 a	3.29 a	0.40 df	0.62 ab	ns	0.15 b	6.8 b	34 ab	50 b
**OPA-MOF**	**7.28 d**	**3.17 a**	**0.44 bcde**	**0.61 b**	**ns**	**0.17 ab**	**8.7 a**	**37 a**	**48 b**
P	10.70 e	2.74 b	0.51 a	0.63 ab	ns	0.17 ab	4.0 c	34 ab	29 c
P+Ca-Ox	11.75 e	2.69 b	0.46 abcde	0.61 b	ns	0.16 ab	3.5 c	35 ab	30 c
N+P	19.30 f	3.18 a	0.46 abcd	0.60 b	ns	0.17 ab	2.7 c	34 ab	31 c
N+P+Ca-Ox	17.74 f	3.13 a	0.43 cdef	0.58 b	ns	0.17 ab	3.1 c	30 b	28 c
*mean*					*0*.*043*				
		**Macronutrient content**					**Micronutrient content**		
**b)**		(mg pot^-1^)					(μg pot^-1^)		
		**N**	**P**	**K**	**Ca**	**Mg**	**Cu**	**Mn**	**Zn**
Control		102.9 AB	15.3 AB	20.9 AB	1.2 AB	5.5 AB	23.8 AB	87 A	183 AB
N		139.8 B	17.1 B	26.7 B	1.8 C	6.7 B	30.4 BCD	170 B	234 BC
Ca-Ox		132.3 AB	18.1 B	23.9 B	1.5 BC	6.6 B	28.4 ABC	113 A	210 B
N+Ca-Ox		92.8 A	11.3 A	17.5 A	1.1 A	4.2 A	19.3 A	97 A	140 A
**OPA-MOF**		**232.3 C**	**31.6 C**	**44.4 C**	**2.9 D**	**59.7 E**	**59.7 E**	**271 C**	**357 C**
P		291.4 C	55.1 D	67.1 D	5.0 E	42.4 DE	42.4 DE	368 D	314 C
P+Ca-Ox		309.1 C	54.4 D	69.9 D	4.8 E	41.1 CDE	41.1 CDE	424 D	364 CD
N+P		610.4 D	88.4 E	114.0 E	9.1 F	51.8 E	51.8 E	650 E	576 E
N+P+Ca-Ox		553.9 D	77.1 E	103.4 E	8.2 E	55.4 E	55.4 E	528 DE	504 DE

Macro- and micronutrient concentrations (part a) and -contents (part b) in wheat grains at maturity from high application rate of various fertilizers. Mean values followed by the same letter are not significantly different at the 5% confidence level. Upper case letters in part b (contents) and for biomass are from analysis of ln-transformed data; ANOVA with n = 4 replicates.

#### Grain nutrient concentrations and contents

Plants that received P in the absence of any N had significantly (p ≤ 0.05) lower grain-N concentrations (around 2.7%) relative to all other treatments (> 3% N; Table 4). Moreover, applications of P without N increased the grain content of all nutrients by 2–3 fold, while concentrations remained stable except for N, Cu, and Zn where concentrations significantly decreased (p ≤ 0.05; Table 4). Plants receiving N alone had significantly lower grain P concentrations than the controls, but grain N concentrations were unaffected (Table 4). Grain Ca concentrations were unaffected in all treatments, and grain Mg concentrations were similar for all treatments except N in absence of P which had reduced Mg levels (Table 4). For P, N, Mg and Ca, grain nutrient contents increased proportionally with biomass (yield). Grain Cu- and Zn concentrations declined significantly (p ≤ 0.05) when P-fertilizer was added, but total content in the grain increased owing to enhanced grain yields.

OPA-MOF treatments significantly increased the content of all nutrients in the grain compared to all treatments that did not receive P, with the exception of Zn. Zn levels did not increase compared to the N-only treatment unless Ca-Ox was added (Table 4). Increased grain nutrient levels tended to be commensurate with total grain yield increases in OPA-MOF treatments, because the concentrations of most nutrients in the grain of OPA-MOF-treated plants were not significantly (p ≤ 0.05) different to plants that did not receive P.

Plants receiving OPA-MOF had lower nutrient contents (except N, Cu and Zn) compared to plants receiving either P alone or N+P combined, which again tended to reflect grain yield differences (Table 4). In OPA-MOF grains, N concentrations were significantly higher (p ≤ 0.05) than P treatments without N; Cu and Zn concentrations were significantly (p ≤ 0.05) higher in the OPA-MOF treatment compared to any P-containing treatments.

### Experiment 2: Soil incubation study

#### Soil nutrient concentrations

**Fig 2:**
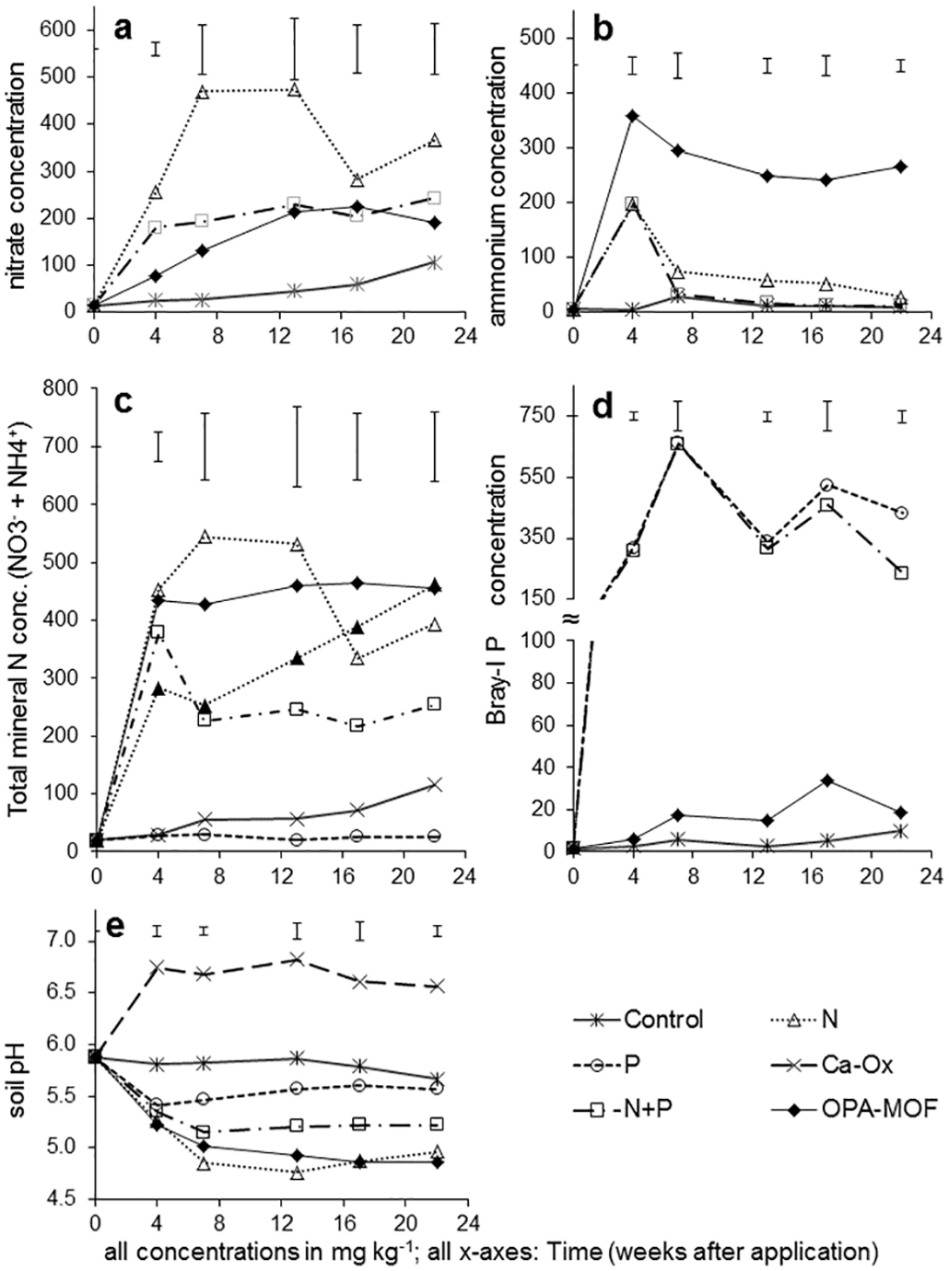
Fertilizer effects on soil nutrients. Effect of fertilizer treatments on soil concentrations of nitrate (a), ammonium (b), total-N (as calculated sum of nitrate- and ammonium-N; c), Bray-I P (d) and soil pH (e) (in mg/kg—y-axis) over time after application at high rate (equivalent to 120 kg N ha^-1^). Error bars above individual graphs represent significance between treatments (standard error of means; s.e.m.) at 95% confidence level for each sampling time (weeks 4, 7, 13, 17 and 22).

N-application as urea (both N alone or in combination with P) resulted in an immediate sharp increase in soil NO_3_^-^- and NH_4_-N to their respective maximum levels in the first 4 weeks after application (Fig 2a and 2b), while treatments without N had no influence on soil N concentrations (Fig 2c). OPA-MOF-application also resulted in a significant increase (p ≤ 0.05) in both N-forms compared to control in the first 4 weeks after application (Fig 2a and 2b). In contrast to the urea treatments, however, OPA-MOF application resulted in a slow and steady nitrate-N increase over the incubation period (maximum of 200 mg kg^-1^ at 17 weeks); while ammonium-N concentration peaked at 4 weeks, at a significantly higher maximum than ammonium-N in the urea treatment. The ammonium-N in the OPA-MOF treatment remained high throughout the entire incubation period (Fig 2a and 2b). Soils receiving N+P had significantly (p ≤ 0.05) lower total mineral N (sum of ammonium-N and nitrate-N) than soils treated with N alone or OPA-MOF (Fig 2c).

Both Bray I- and Bray II-P concentrations were significantly higher where conventional P-fertilizer was added (Fig 2d; data for Bray II-P not shown), whereas treatments without P had no influence on soil P throughout the trial period. OPA-MOF-treated soils showed small but significant (p ≤ 0.05) soil P increases compared to the controls and treatments without P from week 7 onwards (Fig 2d).

#### Soil pH

All treatments caused an immediate and significant (p ≤ 0.05) decline in soil pH compared to controls (Fig 2e). For treatments containing P (+/- N), this pH drop ceased after week 7, with pH stabilising thereafter (for N+P treatments) or increasing again (for P alone). While the total pH decline for both N alone (urea) and OPA-MOF applications was around one pH unit, when P was added to the urea the pH decline was halved. In contrast to the rapid decline caused by the conventional fertilizers, the pH decline from OPA-MOF initially occurred at a slower rate resulting in significantly higher pH from OPA-MOF compared to N alone in week 7. In subsequent weeks, however, the pH was no longer significantly different between N alone and OPA-MOF treatments at 95% confidence level.

## Discussion

### Nutrient dynamics and crop growth in the Ferralsol

Phosphorus deficiency frequently limits crop growth in Ferralsols [24, 29] and P appeared to be the most limiting in the experimental soil given that the addition of P without N increased growth at 6 weeks 3-fold, yet N application in the absence of P did not increase growth at all (Table 3). The soil pH reduction following N-fertilisation (Fig 2e) and the subsequent nitrification reaction (Eq 1)

$$2\text{N}{\text{H}}_{4}^{+}+4{\text{O}}_{2}+4{\text{e}}^{-}\overset{\text{nitrosomonas},\text{nitrobacter}}{\to }\;2\text{N}{\text{O}}_{3}+4{\text{H}}^{+}+2{\text{H}}_{2}\text{O}+12{\text{e}}^{-}$$ (1)

likely exacerbated the problem by increasing P binding to Fe-(oxy)hydroxides in the Ferralsol [30]. The severe degree of plant P-deficiency is further evidenced by the fact that the application of N-fertilizer in the absence of P fertilizer failed to increase plant N-uptake, presumably due to the specific reduction in N-uptake by roots of severely P-deficient plants [31].

However, biomass and grain yield data clearly indicated that the unamended Ferralsol was deficient in N as well as P, and that correction of both deficiencies was required to achieve maximum wheat biomass and grain yields (Tables 3 and 4). Where N and P fertilizer were applied together, N and P concentrations in 6 week-old plant shoots were well above the critical whole shoot concentrations of 2.8% and 3.4% for P and N, respectively [32]. Although N+P treatments provided the highest shoot and grain yields, they also resulted in the greatest mineral N losses from the incubation soils after week 4 (Fig 2c). At 4 weeks ammonium-N concentrations declined sharply in both N-only and N+P treatments (Fig 2b), but this was only matched by concomitant sharp increases in nitrate-N concentrations for N-only treatments (Fig 2a). Ammonia losses are a plausible explanation, and the resulting soil pH of 5.3 in the N + P treatment could account for a loss of N by ammonia volatilisation of up to 10% of applied N at temperatures around 30°C [33, 34]. However, microbial activity in Ferralsols is often P-limited [35], and the addition of P likely increased microbial activity which consequently results in a ‘drawdown’ of N into microbial biomass. As no measurements of soil total N (or microbial biomass N) were made, it is not possible to discern which of these processes made the greatest contribution to the observed reduction in mineral N in the N + P treatment.

Potassium deficiency is also a widely observed nutritional constraint to crop growth on Ferralsols [30], and the shoot K concentrations of 2.53% and 2.08% at 6 WAS in the N + P and N + P + Ca-Ox treatments, respectively, were well below the 3% critical value for maximum biomass yields at this stage [32]. In an acidic soil K^+^ readily exchanges for H^+^, and soil K^+^ concentration changes were found to be commensurate with pH changes (S1 Fig).

### OPA-MOF potential as a novel N- and P-fertilizer

Both the soil incubation study and the plant growth study suggest that the OPA-MOF has potential as an N-fertilizer. Quantities of total mineral N were similar between N-only and OPA-MOF treatments in the first 4 weeks, indicating a substantial release of the urea enclosed in the OPA-MOF’s pores directly after application to the soil by diffusion/leaching of urea from outer pores, as hypothesised (Fig 1). Further, the high extractable ammonium concentrations and low extractable nitrate-N concentrations compared to the urea treatment in the incubation study suggest inhibition of nitrification in the OPA-MOF treatment. There has been a concerted research effort in recent years aimed at reducing the biological oxidation of ammonium to nitrate in soils—including the formulation of N-fertilizer products containing nitrification inhibitors—to reduce agricultural N losses [10]. Two of the most widely used nitrification inhibitors in broad-acre agriculture are dicyanamide (DCD) and 3,4-dimethylpyrazol-phosphate (DMPP) (36). Unfortunately, both products are less effective at higher temperatures [36, 37], and in the case of DMPP, lose efficacy after 40–50 days [36]. The fact that soil-extractable ammonium concentrations remained high for >100 days in temperatures > 30°C in the OPA-MOF treatment (Fig 2b) suggests that OPA-MOF fertilizers have strong potential to increase N use efficiency in agriculture. [38–40]One mechanism by which high soil-extractable ammonia concentrations were maintained in the OPA-MOF treatment may be a high binding affinity of the OPA-MOF for cations such as ammonia, which reduced the amount of labile ammonia in the soil solution available for nitrification. A further potential mechanism is the presence of Fe(II) in the Fe-P-complex and subsequent redox processes, such as oxidation of Fe(II) to Fe(III) coupled with reduction of Mn(IV) to Mn(II), which are discussed in detail below.

Significant increases (p ≤ 0.05) in shoot and plant biomass and grain yields above the nil control (Tables 3 and 4), and small but significant increases in Bray 1-P concentrations in the soil incubation study (Fig 2d) provide evidence that a portion of the structural-P in the OPA-MOF became bioavailable, despite a continuous pH decline associated with the OPA-MOF application. The observed biomass and grain yield increases in the OPA-MOF treatment cannot be attributed to an N-effect, because N application alone did not result in biomass and yield increases (Tables 3 and 4). Furthermore, significantly increased shoot N concentrations (Tables 3 and 4) suggest that the OPA-MOF treatments overcame the P-deficiency and induced N-uptake limitation that was observed in N-only treatments. The data therefore supported our hypothesis that microbiological mineralisation of the OPA-MOF in soil provides a slow release of plant available P. Unfortunately, the timing and/or rate of P release was insufficient to prevent P deficiency and consequent biomass and grain yield losses in the OPA-MOF treatment compared with +P control treatments.

The P-uptake from the OPA-MOF observed at 6 WAS (Table 3) may be the result of roots proliferating around the OPA-MOF granules with subsequent mobilisation of the structural P in the OPA-MOF framework. Alternatively, structural faults on crystalline surfaces (which are common) may have led to exposed, less strongly bound phosphates being made bio-available, which usually is the case in “fresh” minerals [34]. We cannot discount the possibility that plant exudates (organic acids) improved P-bioavailability, or even the prospect of dissolved oxalate originating from the OPA-MOF enhancing phosphate bioavailability. Despite these possibilities, the fact remains that P-availability was the limiting factor in the OPA-MOF-system.

We hypothesised that P-bioavailability from the Fe-P in the OPA-MOF fertilizer in the acid soil would be promoted by the alkalinity generated from the bacterial reduction of the oxalate linker molecules (Fig 1). Regardless of whether alkalinity increased in micro-environments surrounding the molecules, the net effect of the OPA-MOF-fertilizer was a significant and continual decline in soil pH over time (Fig 2e), which would have a negative impact on P-bioavailability.

In contrast to N-only treatments, where lower soil pH resulted from nitrification, the pH decline in the OPA-MOF treatment (where nitrification was inhibited) is likely associated with the redox-induced oxidation of structural Fe^2+^ in the OPA-MOF (Eq 2).

$$4\text{F}{\text{e}}_{2}^{+}+{\text{O}}_{2}+10{\text{H}}_{2}\text{O}\;\to 4\text{Fe}{\left(\text{OH}\right)}_{3}+8{\text{H}}^{+}+4{\text{e}}^{-}\;({\text{E}}^{0}\;=\;0.77\text{V})$$ (2)

Physical characterisation of the OPA-MOF suggests that about a third of the Fe in the OPA-MOF is present as Fe^2+^ (Anstoetz, unpublished data). The presence of the Fe(II) in the OPA-MOF structures is supported by others (e.g. [41–45] Hence, because of a higher redox-potential of Fe(II) oxidation to Fe(III) compared with NH_4_^+^ nitrification, Fe(II) oxidation is the preferred reaction, resulting in release of 2H^+^-ions for each oxidised Fe-atom [46] Thus, in addition to driving acidification, the process also limits nitrification.

Oxidation of Fe(II) supplies electrons to allow for reduction of a redox-partner which could explain the observed increase in Mn-bioavailability in OPA-MOF treatments (S1 Fig). Manganese (IV) reduction to plant-available Mn(II) is exceedingly favourable under Fe(II) oxidation[46]. The importance of redox chemistry in conjunction with pH in plant soil systems has previously been discussed in detail by Husson [47]. Strongly increased shoot-Mn concentration for plants growing in the OPA-MOF treatments are of concern because of potential Mn toxicity. However, no plant Mn toxicity symptoms were observed.

While the results from this study are promising, more work on OPA-MOFs as potential novel fertilizers is required. For example, incorporating different Fe-species (Fe(III):Fe(II) ratios), or altogether different central ions (e.g. Zn, Co, and Mo) into the framework structure may limit the soil acidifying effect of OPA-MOF, and consequent Mn(IV) reduction. Alternatively, in alkaline soils the acidity generated from the ferrous-oxidation may increase the mobilisation of apatite-bound P, which tends to be acid soluble [48, 49], and the OPA-MOF may also act as a source of Fe to plants in alkaline calcareous soils where soil Fe is often unavailable to plants due to complexation. Research on alternative central cations and the effectiveness of OPA-MOFs in alkaline soils is continuing in our lab.

## Conclusions

The immediate release of mineral N from the OPA-MOF and subsequent retention of the bulk of this N as an ammonium form for over 100 d, with temperatures reaching over 30°C, suggests that OPA-MOF-type fertilizers have the potential to be used as novel enhanced efficiency N-fertilizers. The release rate and/or bioavailability of P from the OPA-MOF limited plant growth in the acid soil, and further investigation into alternative central cations (other than Fe), and the impact of OPA-MOFs in alkaline soils, is required.

## Supporting Information

S1 Fig Soil K concentrations. Influence of fertilizer treatment on available soil K concentrations in an incubation study of 22 weeks; error bars represent significance between treatments (standard error of means) at the 95% confidence level; where two treatments appear combined, there were no significant differences at any one sampling time point at the 95% confidence level.
(DOCX)

S2 Fig Soil Mn concentrations. Influence of fertilizer treatment on soil Mn concentrations in an incubation study of 22 weeks; error bars above the graph represent significance between treatments (standard error of means; s.e.m.) at the 95% confidence level; where two treatments appear combined, there were no significant differences at any one sampling time point at the 95% confidence level; ANOVA with n = 4 replicates.
(DOCX)

S1 File Tillers and leaves. This file contains original data from the pot trials showing the plant properties “tillers” and “leaves”.
(XLSX)

S2 File 1000-grain weight. This file contains statistical data for significance test of 1000-grain-weights.
(DOCX)

S1 Table Nutrients in shoot tissue from low treatment rate. Macro- and micronutrient concentrations (part a) and -contents (part b) in shoot tissue of six-week-old wheat plants from low application rate of various fertilizers; mean values followed by the same letter are not significantly different at the 5% confidence level; upper case letters in part b (contents) and for biomass are from analysis of ln-transformed data; ANOVA with n = 4 replicates.
(DOCX)

S2 Table Nutrients in grains from low treatment rate. Macro- and micronutrient concentrations (part a) and -contents (part b) in wheat grains at maturity from low application rate of various fertilizers; mean values followed by the same letter are not significantly different at the 5% confidence level; upper case letters in part b (contents) and for biomass are from analysis of ln-transformed data; ANOVA with n = 4 replicates.
(DOCX)
