# Supplementary material for: Novel Applications for Oxalate-Phosphate-Amine Metal-Organic-Frameworks (OPA-MOFs): Can an Iron-Based OPA-MOF Be Used as Slow-Release Fertilizer?
Source: PLoS One. 2015 Dec 3;10(12):e0144169. doi: 10.1371/journal.pone.0144169 (PMC4669090; doi:10.1371/journal.pone.0144169)
Supplement: S2 Fig — Influence of fertilizer treatment on soil Mn concentrations in an incubation study of 22 weeks; error bars above the graph represent significance between treatments (standard error of means; s.e.m.) at the 95% confidence level; where two treatments appear combined, there were no significant differences at any one sampling time point at the 95% confidence level; ANOVA with n = 4 replicates. (DOCX) [file pone.0144169.s002.docx]

**Novel applications for oxalate-phosphate-amine metal-organic-frameworks (OPA-MOFs): can an iron-based OPA-MOF be used as slow release fertilizer?**

Manuela Anstoetz^*1^, Terry Rose^3^, Malcolm Clark^1,2^, Lachlan H. Yee^1,2^, Carolyn Raymond^3^, Tony Vancov^3,4^

* Corresponding author, email: manuela.anstoetz@scu.edu.au

^1^ School of Environment, Science and Engineering, Southern Cross University, Lismore NSW 2480, Australia

^2^ Marine Ecology Research Centre, School of Environment, Science and Engineering, Southern Cross University, Lismore NSW 2480, Australia

^3^ Southern Cross Plant Science, Southern Cross University, Lismore NSW 2480, Australia

^4^ NSW Department of Primary Industries, Wollongbar Primary Industries Institute, Wollongbar, NSW, 2480, Australia

**S2 Fig**
