# Supplementary material for: Novel Applications for Oxalate-Phosphate-Amine Metal-Organic-Frameworks (OPA-MOFs): Can an Iron-Based OPA-MOF Be Used as Slow-Release Fertilizer?
Source: PLoS One. 2015 Dec 3;10(12):e0144169. doi: 10.1371/journal.pone.0144169 (PMC4669090; doi:10.1371/journal.pone.0144169)
Supplement: S2 File — This file contains statistical data for significance test of 1000-grain-weights. (DOCX) [file pone.0144169.s004.docx]

Analysis of variance

Variate: weight_1000_seeds

Source of variation d.f. s.s. m.s. v.r. F pr.

Block 3 75.79 25.26 1.79 0.176

TMT 8 149.79 18.72 1.33 0.279

Residual 24 339.13 14.13

Total 35 564.72

*Message: the following units have large residuals.*

*units* 30 8.40 approx. s.e. 3.07

*units* 31 -8.49 approx. s.e. 3.07

Tables of means

Variate: weight_1000_seeds

Grand mean 30.17

Block 1 2 3 4

30.53 27.71 31.35 31.09

TMT C MH ML NH NL NOXH NOXL

30.52 32.68 32.16 28.41

TMT NPH NPL NPOXH NPOXL OXH OXL PH

28.08 31.32 32.18 26.42

TMT PL POXH POXL

29.78

Standard errors of differences of means

Table Block TMT

rep. 9 4

d.f. 24 24

s.e.d. 1.772 2.658

1190 DELETE [REDEFINE=yes] _mean, _rep, _var, _resid, _rdf, _scode
 1191 AKEEP [FACTORIAL=9] TMT; MEAN=_mean; REP=_rep; VARIANCE=_var; RTERM=_resid; STATUS=_scode
 1192 IF _scode.IN.!(1,2)
 1193 AKEEP [FACTORIAL=9] #_resid; DF=_rdf
 1194 AMCOMPARISON [PRINT=letter; METHOD=duncan; DIRECTION=descending; PROB=0.05] TMT

Duncan's multiple range test

TMT

Mean

MH 32.68 a

OXH 32.18 a

NH 32.16 a

NPOXH 31.32 a

C 30.52 a

POXH 29.78 a

NOXH 28.41 a

NPH 28.08 a

PH 26.42 a

1195 ELSE
 1196 PRINT !t('Multiple comparisons available only if all components of the term',\
 1197 'are estimated with equal efficiency and in the same stratum.');\
 1198 JUST=left
 1199 ENDIF
