# Supplementary material for: Novel Applications for Oxalate-Phosphate-Amine Metal-Organic-Frameworks (OPA-MOFs): Can an Iron-Based OPA-MOF Be Used as Slow-Release Fertilizer?
Source: PLoS One. 2015 Dec 3;10(12):e0144169. doi: 10.1371/journal.pone.0144169 (PMC4669090; doi:10.1371/journal.pone.0144169)
Supplement: S1 Table — Macro- and micronutrient concentrations (part a) and -contents (part b) in shoot tissue of six-week-old wheat plants from low application rate of various fertilizers; mean values followed by the same letter are not significantly different at the 5% confidence level; upper case letters in part b (contents) and for biomass are from analysis of ln-transformed data; ANOVA with n = 4 replicates. (DOCX) [file pone.0144169.s005.docx]

**Novel applications for oxalate-phosphate-amine metal-organic-frameworks (OPA-MOFs): can an iron-based OPA-MOF be used as slow release fertilizer for wheat crops?**

Manuela Anstoetz^*1^, Terry Rose^3^, Malcolm Clark^1,2^, Lachlan H. Yee^1,2^, Carolyn Raymond^3^, Tony Vancov^3,4^

* Corresponding author, email: manuela.anstoetz@scu.edu.au

^1^ School of Environment, Science and Engineering, Southern Cross University, Lismore NSW 2480, Australia

^2^ Marine Ecology Research Centre, School of Environment, Science and Engineering, Southern Cross University, Lismore NSW 2480, Australia

^3^ Southern Cross Plant Science, Southern Cross University, Lismore NSW 2480, Australia

^4^ NSW Department of Primary Industries, Wollongbar Primary Industries Institute, Wollongbar, NSW, 2480, Australia

S1 Table. Nutrients in shoot tissue from low treatment rate.

| **Treatment** | **Biomass** | **Macronutrient concentration** | | | | | **Micronutrient concentration** | | |
| --- | --- | --- | --- | --- | --- | --- | --- | --- | --- |
|  | (mg pot^-1^) | (%) | | | | | (mg kg^-1^) | | |
| **a)** |  | **N** | **P** | **K** | **Ca** | **Mg** | **Cu** | **Mn** | **Zn** |
| Control | 174.5 A | 3.66 b | 0.15 ab | 5.07 cd | 0.71 b | 0.27 ab | 12.0 c | 88 d | 38 c |
| N | 197.5 AB | 3.96 bc | 0.13 a | 4.90 cd | 0.75 b | 0.26 ab | 9.9 b | 154 f | 48 e |
| Ca-Ox | 186.2 A | 3.58 b | 0.15 ab | 4.93 cd | 0.60 a | 0.24 a | 8.0 a | 74 c | 37 c |
| N+Ca-Ox | 184.2 A | 4.03 bcd | 0.16 b | 5.19 d | 0.72 b | 0.25 ab | 14.0 d | 122 e | 43 d |
| OPA-MOF | 236.2 B | 4.55 de | 0.23 c | 4.80 c | 0.73 b | 0.28 b | 10..0 b | 152 f | 51 f |
| P | 502.0 C | 2.36 a | 0.43 d | 3.80 b | 0.61 a | 0.23 a | 8.0 a | 73 c | 22 a |
| P+Ca-Ox | 466.2 C | 2.30 a | 0.45 d | 3.76 b | 0.69 b | 0.25 ab | 8.0 a | 61 b | 20 a |
| N+P | 848.8 D | 4.61 e | 0.51 e | 2.30 a | 0.95 c | 0.49 c | 10.0 b | 61 b | 32 b |
| N+P+Ca-Ox | 807.5 D | 4.22 cde | 0.52 e | 2.33 a | 0.98 c | 0.49 c | 9.2 b | 50 a | 33 b |
|  | **Biomass** | **Macronutrient content** | | | | | **Micronutrient content** | | |
|  | (mg pot^-1^) | (mg pot^-1^) | | | | | (µg pot^-1^) | | |
| **b)** |  | **N** | **P** | **K** | **Ca** | **Mg** | **Cu** | **Mn** | **Zn** |
| Control | 174.5 A | 6.39 A | 0.26 A | 8.85 A | 1.24 AB | 0.47 A | 2.09 B | 15.36 A | 6.63 A |
| N | 197.5 AB | 7.82 A | 0.26 A | 9.68 AB | 1.48 BC | 0.51 A | 4.24 D | 30.42 CD | 9.48 BC |
| Ca-Ox | 186.2 A | 6.67 A | 0.28 A | 9.18 A | 1.12 A | 0.45 A | 1.49 A | 13.78 A | 6.89 A |
| N+Ca-Ox | 184.2 A | 7.43 A | 0.30 A | 9.56 Ab | 1.33 AB | 0.46 A | 2.58 C | 22.48 B | 7.92 AB |
| OPA-MOF | 236.2 B | 10.75 B | 0.54 B | 11.32 B | 1.73 C | 0.66 B | 2.36 BC | 35.91 CDE | 12.05 D |
| P | 502.0 C | 11.84 b | 2.19 C | 19.03 C | 3.09 D | 1.18 C | 4.02 D | 36.82 DE | 11.06 CD |
| P+Ca-Ox | 466.2 C | 10.70 B | 2.07 C | 17.43 C | 3.18 D | 1.16 C | 3.73 D | 28.53 C | 9.32 BC |
| N+P | 848.8 D | 38.62 C | 4.31 D | 19.39 C | 9.97 E | 4.13 D | 8.42 E | 51.52 F | 27.19 E |
| N+P+Ca-Ox | 807.5 D | 33.78 C | 4.14 D | 18.37 C | 9.97 E | 3.95 D | 7.49 E | 41.03 E | 26.45 E |

Macro- and micronutrient concentrations (part a) and -contents (part b) in shoot tissue of six-week-old wheat plants from low application rate of various fertilizers; mean values followed by the same letter are not significantly different at the 5% confidence level; upper case letters in part b (contents) and for biomass are from analysis of ln-transformed data; ANOVA with n=4 replicates
