# Supplementary material for: Novel Applications for Oxalate-Phosphate-Amine Metal-Organic-Frameworks (OPA-MOFs): Can an Iron-Based OPA-MOF Be Used as Slow-Release Fertilizer?
Source: PLoS One. 2015 Dec 3;10(12):e0144169. doi: 10.1371/journal.pone.0144169 (PMC4669090; doi:10.1371/journal.pone.0144169)
Supplement: S2 Table — Macro- and micronutrient concentrations (part a) and -contents (part b) in wheat grains at maturity from low application rate of various fertilizers; mean values followed by the same letter are not significantly different at the 5% confidence level; upper case letters in part b (contents) and for biomass are from analysis of ln-transformed data; ANOVA with n = 4 replicates. (DOCX) [file pone.0144169.s006.docx]

**Novel applications for oxalate-phosphate-amine metal-organic-frameworks (OPA-MOFs): can an iron-based OPA-MOF be used as slow release fertilizer for wheat crops?**

Manuela Anstoetz^*1^, Terry Rose^3^, Malcolm Clark^1,2^, Lachlan H. Yee^1,2^, Carolyn Raymond^3^, Tony Vancov^3,4^

* Corresponding author, email: manuela.anstoetz@scu.edu.au

^1^ School of Environment, Science and Engineering, Southern Cross University, Lismore NSW 2480, Australia

^2^ Marine Ecology Research Centre, School of Environment, Science and Engineering, Southern Cross University, Lismore NSW 2480, Australia

^3^ Southern Cross Plant Science, Southern Cross University, Lismore NSW 2480, Australia

^4^ NSW Department of Primary Industries, Wollongbar Primary Industries Institute, Wollongbar, NSW, 2480, Australia

S2 Table. Nutrients in grains from low treatment rate.

| **Treatment** | **Biomass** | **Macronutrient concentration** | | | | | **Micronutrient concentration** | | |
| --- | --- | --- | --- | --- | --- | --- | --- | --- | --- |
|  | (mg pot^-1^) | (%) | | | | | (mg kg^-1^) | | |
| **a)** |  | **N** | **P** | **K** | **Ca** | **Mg** | **Cu** | **Mn** | **Zn** |
| Control | 3.00 A | 3.43 a | 0.50 a | 0.70 a | ns | 0.18 a | 7.7 a | 29 b | 60 a |
| N | 3.37 A | 3.27 a | 0.43 bc | 0.67 ab | ns | 0.16 ab | 7.2 ab | 39 ab | 58 a |
| Ca-Ox | 3.24 A | 3.37 a | 0.48 ab | 0.65 abc | ns | 0.16 ab | 7.8 a | 29 b | 55 a |
| N+Ca-Ox | 4.42 AB | 3.30 a | 0.38 cd | 0.59 cd | ns | 0.15 b | 6.7 ab | 28 b | 50 a |
| OPA-MOF | 6.14 BC | 3.25 a | 0.45 ab | 0.63 bc | Ns | 0.17 ab | 7.1 ab | 37 a | 55 a |
| P | 10.28 CD | 2.57 c | 0.42 bcd | 0.58 cd | ns | 0.17 ab | 4.2 cd | 34 ab | 28 b |
| P+Ca-Ox | 10.15 CD | 2.54 c | 0.43 bc | 0.58 cd | ns | 0.15 b | 5.4 bc | 35 b | 28 b |
| N+P | 13.81 D | 2.91 b | 0.36 d | 0.57 cd | ns | 0.16 b | 3.3 d | 34 b | 27 b |
| N+P+Ca-Ox | 14.15 D | 2.97 b | 0.39 cd | 0.54 d | ns | 0.16 b | 3.6 cd | 30 b | 29 b |
| *mean* |  |  |  |  | *0.041* |  |  |  |  |
|  | **Biomass** | **Macronutrient content** | | | | | **Micronutrient content** | | |
|  | (mg pot^-1^) | (mg pot^-1^) | | | | | (µg pot^-1^) | | |
| **b)** |  | **N** | **P** | **K** | **Ca** | **Mg** | **Cu** | **Mn** | **Zn** |
| Control | 3.00 A | 102.9 A | 15.3 A | 20.9 A | 1.2 A | 5.5 A | 23.8 AB | 87.2 A | 182.9 A |
| N | 3.37 A | 109.6 A | 14.4 A | 21.9 A | 1.4 A | 5.4 A | 24.5 A | 111.9 A | 188.2 A |
| Ca-Ox | 3.24 A | 108.0 A | 16.0 A | 21.6 A | 1.4 A | 5.1 A | 25.8 AB | 90.8 A | 182.7 A |
| N+Ca-Ox | 4.42 AB | 143.4 AB | 16.1 A | 25.4 A | 1.7 A | 6.3 A | 27.3 AB | 127.0 A | 203.0 A |
| OPA-MOF | 6.14 BC | 199.0 BC | 27.4 B | 38.3 B | 2.5 B | 10.5 B | 43.5 BC | 214.1 B | 326.0 B |
| P | 10.28 CD | 263.0 CD | 43.3 BC | 59.5 BC | 4.4 BC | 17.3 BC | 42.4 BC | 343.9 B | 289.8 AB |
| P+Ca-Ox | 10.15 CD | 256.9 CD | 43.9 BC | 59.4 BC | 4.1 BC | 15.3 BC | 54.8 C | 285.1 B | 287.2 AB |
| N+P | 13.81 D | 400.4 D | 50.1 BC | 79.2 C | 6.0 C | 21.6 C | 51.8 C | 398.6 B | 372.7 B |
| N+P+Ca-Ox | 14.15 D | 419.8 D | 55.0 B | 76.4 C | 5.8 C | 22.4 C | 51.7 C | 404.3 B | 414.2 B |

Macro- and micronutrient concentrations (part a) and -contents (part b) in wheat grains at maturity from low application rate of various fertilizers; mean values followed by the same letter are not significantly different at the 5% confidence level; upper case letters in part b (contents) and for biomass are from analysis of ln-transformed data; ANOVA with n=4 replicates
